# Patients with chronic periodontitis present increased risk for primary Sjögren syndrome: a nationwide population-based cohort study

**DOI:** 10.7717/peerj.5109

**Published:** 2018-06-20

**Authors:** Tai-Chen Lin, Chien-Fang Tseng, Yu-Hsun Wang, Hui-Chieh Yu, Yu-Chao Chang

**Affiliations:** 1 Department of Dentistry, Chung Shan Medical University Hospital, Taichung, Taiwan; 2 School of Dentistry, Chung Shan Medical University, Taichung, Taiwan; 3 Department of Medical Research, Chung Shan Medical University Hospital, Taichung, Taiwan

**Keywords:** Chronic periodontitis, Primary Sjögren syndrome, Nationwide population, Cohort study, Taiwan

## Abstract

Many reports have mentioned the association between chronic periodontitis (CP) and primary Sjögren syndrome (pSS). However, no cohort study has been performed for the risk of pSS in patients with CP. In this study, we evaluated the risk of pSS from CP exposure in a nationwide population-based cohort study in Taiwan. We studied the claims data of Taiwanese population from 2001 to 2012. We identified 76,765 patients with CP from the National Health Insurance Database in Taiwan. We also selected 76,765 controls that were randomly frequency matched by age, sex, and index year from the general population. We analyzed the risk of pSS by using Cox proportional hazards regression models including sex, age, and comorbidities. In this study, 76,765 patients with CP (mean age: 40.8 years) and 76,765 controls (mean age: 41.0 years) were followed-up for 8.54 and 8.49 years, respectively. A total of 869 cases of pSS were identified in CP cohort and 483 cases in non-CP cohort. Multivariate Cox regression analysis indicated that the incidence rate of pSS was significantly higher in CP cohort than those who in non-CP cohort (adjusted HR: 1.79, 95% CI [1.60–2.00]). Taken together, this nationwide retrospective cohort study demonstrated that the risk of pSS was significantly higher in patients with CP than in the general population. The association between CP and pSS was significant in the female group.

## Introduction

Chronic periodontitis (CP) is the most common periodontal disease which is related to the chronic accumulation of bacterial biofilm leading to host-mediated slowly progressive destruction of periodontium. CP is the primary risk factor for tooth loss in adults (Agerholm, 2001). Recently, studies have also shown that CP is a risk factor for many systemic diseases including cardiovascular disease (Dietrich et al., 2013), diabetes mellitus (Borgnakke et al., 2013), and rheumatoid arthritis (Tang et al., 2017). The possible mechanisms involving the modulations of inflammatory pathway and systemic immunity are associated with a bacterial challenge and represent a portal of entry for periodontal pathogens, bacterial endotoxins, and pro-inflammatory cytokines (Ebersole et al., 2017).

Primary Sjögren’s syndrome (pSS) is a systemic disease characterized by chronic autoimmune reaction with progressive lymphocytic infiltration of salivary and lacrimal glands leading to dry eyes and dry mouth (Ramos-Casals & Font, 2005; Ramos-Casals et al., 2012). Secondary Sjögren’s syndrome occurs in the presence of other autoimmune diseases such as rheumatoid arthritis or systemic lupus erythematosus. The prevalence of pSS is higher among female than among in male (Qin et al., 2015). The incidence rate of pSS was 10.6 per 100,000 person-years in Taiwan (Yu et al., 2013).

The increasing prevalence of CP was found in pSS patients with compromised salivary flow and consequent higher dental plaque index (Boutsi et al., 2000; Pers et al., 2005). Contrarily, Lugonja et al. (2016) reported no increase in the prevalence of CP in pSS patients. However, no cohort study has been conducted to evaluate the risk of pSS in patients with CP in a large population. Therefore, the association between CP and pSS still needs further investigation.

The Taiwan National Health Insurance (NHI) program is a mandatory health insurance program that began in 1995 and has provided health care up to 99.9% population in 2014 (National Health Insurance Administration, 2014). The National Health Insurance Research Database (NHIRD) was established in 1997 for scientific and research studies. This database has been used for many longitudinal epidemiological studies for assessment the burden of periodontitis (Chen, Wu & Chang, 2017a, 2017b; Yu et al., 2017, 2018; Su et al., 2017). Therefore, this study assessed the risk of pSS in a large, nationally representative, population-based cohort of patients with CP in Taiwan.

## Methods

### Data source

The Longitudinal Health Insurance Database 2010 (LHID2010) was used for this cohort study. This database provides scrambled patient identification number, date of birth, sex, diagnostic codes in the format of the International Classification of Disease, Revision 9, Clinical Modification (ICD-9-CM) code, and the date of visit to medical institutes as described previously (Chen, Wu & Chang, 2017b; Su et al., 2017).

### Exposure of CP

After being approved by the Chung Shan Medical University Hospital institutional review board (CS2-15071), we identified the ambulatory patients for dental visit with newly diagnosed CP (ICD-9-CM code: 523.4) from 2001 to 2012 as a newly onset CP cohort. The first-time CP diagnosis served as the index date. To ensure the accuracy of CP diagnosis, only patients with at least three outpatient service claims were recruited.

Subjects without periodontitis were randomly selected from the data set and identified as periodontally healthy controls. The comparison group included the participations who were never diagnosed with CP from 2001 to 2012. In order to reduce the confounding bias, we used propensity score matching to select controls. Propensity score of participants which predicted the probability of CP exposure for participants was estimated by logistic regression modeling. In addition to traditional confounding factors, chronic insomnia (ICD-9-CM, code 780.52) was first included to assess as recently reported as an important risk factor of pSS (Kok et al., 2016). The predictors involved birth year, sex, and co-morbidities at baseline. The 1:1 matched comparisons were selected with the same propensity score in as exposure subject. Patients diagnosed as having periodontitis before 2001 were excluded.

### Primary Sjögren syndrome event

The diagnosis and code (ICD-9-CM 710.2) of pSS were according to the European classification criteria for Sjögren syndrome in 2002 (Qin et al., 2015). All participants were followed-up from index date to the date of the primary outcome, withdrawal from the NHI programme or the end of 2013, whichever came first. In addition, we excluded patients with comorbidities such as systemic lupus erythematous (ICD-9-CM 710.0), rheumatoid arthritis (ICD-9-CM 714), ankylosing spondylitis (ICD-9-CM 720.0), polymyositis (ICD-9-CM 710.4), and dermatomyositis (ICD-9-CM 710.3) to limit our study sample as pSS during the observation period.

### Statistical analysis

The Student’s *t* test and Chi-square test were used to analyze the difference of continuous and categorical variables, respectively. The Cox proportional hazard models were applied to estimate the hazard ratios of CP. All statistical analyses were performed with the SPSS version 18 (SPSS, Chicago, IL, USA), and the significant level was 0.05.

## Results

As shown in Table 1, we enrolled 76,765 patients with CP and 76,765 subjects without CP. The two groups had similar mean age and sex ratio because of the frequency matching for age and sex. There were also no income and urbanization variation (*p* > 0.05). The percentage of comorbidities hypertension, hyperlipidemia, chronic insomnia, and CCI showed no significant differences between patients with CP and the healthy controls (*p* > 0.05).

**Table 1:** Demographic data.

	Chronic periodontitis (*N* = 76,765)		Control (*N* = 76,765)		*p*-value
	*n*	%	*n*	%	
Age					0.991
<20	8,271	10.8	8,252	10.7	
20–39	29,359	38.2	29,333	38.2	
40–64	32,665	42.6	32,725	42.6	
≧65	6,470	8.4	6,455	8.4	
Mean ± SD	40.8 ± 16.3		41.0 ± 16.2		0.011*
Gender					0.613
Female	40,525	52.8	40,624	52.9	
Male	36,240	47.2	36,141	47.1	
Monthly income					0.772
<NT $20,000	37,636	49.0	37,609	49.0	
NT $20,000–NT $40,000	21,188	27.6	21,301	27.7	
>NT $40,000	17,941	23.4	17,855	23.3	
Urbanization					0.857
Urban	51,719	67.4	51,748	67.4	
Suburban	20,704	27.0	20,725	27.0	
Rural	4,342	5.7	4,292	5.6	
Hypertension	9,421	12.3	9,472	12.3	0.692
Hyperlipidemia	5,652	7.4	5,670	7.4	0.860
Chronic insomnia	1,755	22.9	17,495	22.8	0.738
CCI^†^					0.819
0	58,417	76.1	58,342	76.0	
1	15,107	19.7	15,136	19.7	
≧2	3,241	4.2	3,287	4.3	

**Notes:**

Demographic data of matched study cohorts.

The Student’s *t* test and Chi-squared test were used to test the difference of continuous and categorical variables, respectively.

† Charlson comorbidity index.

* *p* < 0.05.

**Figure 1:**
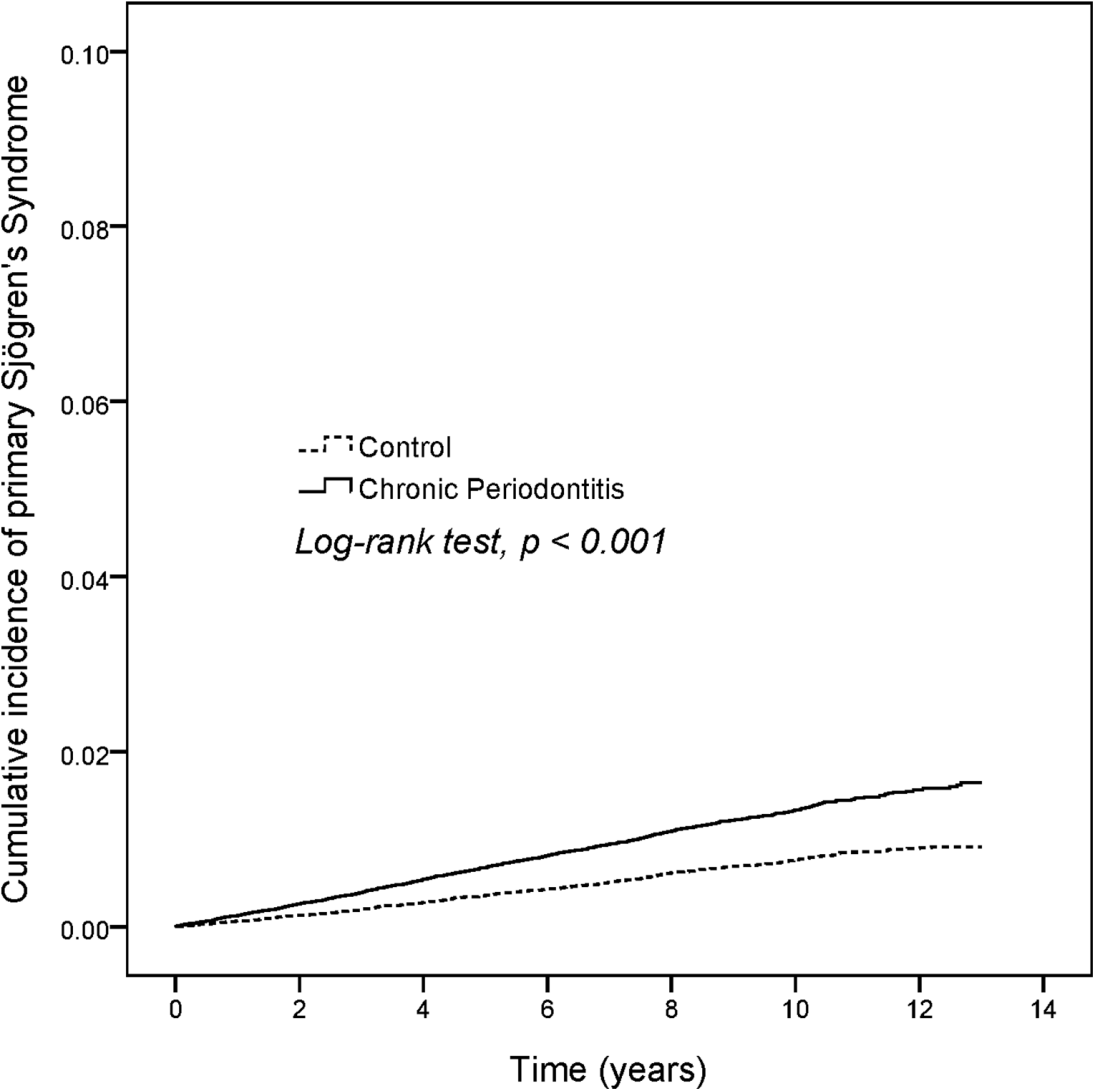
KM plot.

The newly diagnosed pSS patients were 869 in CP group and 483 individuals in non-CP group. The incidence density rates of pSS in non-CP group was only 0.7 per 1,000 person-years (Table 2), but the incidence density rates was about 1.86-fold higher in CP group than in non-CP group (1.3 per 1,000 person-years). Figure 1 shows the cumulative curve of the pSS incidence and reveals that the curve of CP patients was significantly higher than the curve of control subjects (log rank test, *p* < 0.001). After adjustment of sex, age, monthly income, urbanization, and comorbidities, CP patients showed a 1.79-fold increased risk of pSS compared with non-CP patients (HR 1.79, 95% CI [1.60–2.00]). However, there was no significant risk for pSS with income, urbanization, and hypertension in the adjusted model.

**Table 2:** Risk factors.

	No. of event	Observed person-years	ID	Crude HR	95% C.I.		Adjusted HR^¶^	95% CI	
					Lower	Upper		Lower	Upper
Chronic periodontitis									
No	483	651,552	0.7	1			1		
Yes	869	655,566	1.3	1.79**	1.60	2.00	1.79**	1.60	2.00
Age									
<20	53	135,583	0.4	1			1		
20–39	339	518,847	0.7	1.67**	1.25	2.23	1.56**	1.16	2.10
40–64	747	549,571	1.4	3.48**	2.63	4.60	2.82**	2.11	3.78
≧65	213	103,117	2.1	5.28**	3.91	7.14	3.67**	2.66	5.05
Gender									
Male	352	615,103	0.6	1			1		
Female	1,000	692,015	1.4	2.52**	2.24	2.85	2.55**	2.25	2.88
Monthly income									
<NT $20,000	677	639,530	1.1	1			1		
NT $20,000–NT $40,000	384	359,006	1.1	1.01	0.89	1.15	0.94	0.82	1.07
>NT $40,000	291	308,582	0.9	0.89	0.78	1.02	1.02	0.88	1.18
Urbanization									
Urban	903	879,933	1.0	1			1		
Suburban	381	354,024	1.1	1.05	0.93	1.18	1.07	0.94	1.20
Rural	68	73,161	0.9	0.91	0.71	1.16	0.86	0.67	1.11
Hypertension	266	149,842	1.8	1.89**	1.65	2.16	0.99	0.84	1.15
Hyperlipidemia	176	86,980	2.0	2.10**	1.79	2.46	1.24*	1.04	1.47
Chronic insomnia	634	304,042	2.1	2.91**	2.62	3.24	2.08**	1.86	2.32
CCI^†^									
0	839	999,322	0.8	1			1		
1	421	256,471	1.6	1.95**	1.74	2.20	1.38**	1.21	1.56
≧2	92	51,325	1.8	2.14**	1.72	2.65	1.30*	1.03	1.62

**Notes:**

Risk factor analysis of primary Sjögren’s syndrome development.

† Charlson comorbidity index; ID: Incidence density, per 1,000 person-years.

¶ Adjusted for age, gender, monthly income, urbanization, hypertension, hyperlipidemia, chronic insomnia, and Charlson comorbidity index.

* *p* < 0.05.

** *p* < 0.01.

In the stratified follow-up duration analysis, the risk of pSS was significantly higher by 1.87, 1.66, and 1.65 fold during the first seven years, seven to 10 years, the subsequent 10 years, respectively, in CP cohort compared with in non-CP cohort (Table 3).

**Table 3:** Hazard ratios.

Follow-up duration (years)	Chronic periodontitis		Control		HR^†^	95% CI	
	No. of event	ID	No. of event	ID		Lower	Upper
<7 yrs	649	5.5	347	2.9	1.87**	1.64	2.13
7–10 yrs	163	0.9	102	0.5	1.66**	1.30	2.13
≧10 yrs	57	0.2	34	0.1	1.65*	1.08	2.53

**Notes:**

Hazard ratios of primary Sjögren’s syndrome stratified by follow-up duration.

ID: Incidence density, per 1,000 person-years.

† Adjusted for age, gender, monthly income, urbanization, hypertension, hyperlipidemia, chronic insomnia, and Charlson comorbidity index.

* *p* < 0.05.

** *p* < 0.01.

The mean follow-up duration and time to pSS between the CP and the non-CP group was shown in Table 4. The mean follow-up duration of pSS was 8.54 years and 8.49 years for the CP and non-CP group, respectively. The mean time to pSS was 4.75 and 4.98 years for the CP and non-CP group, respectively.

**Table 4:** Time to event.

	Chronic periodontitis (*N* = 76,765)	Control (*N* = 76,765)	*p*-value
Follow-up duration (years)	8.54 ± 3.08	8.49 ± 3.10	0.001**
Time to event (years), *N* = 1,352	4.75 ± 3.06	4.98 ± 3.03	0.190

**Notes:**

Track time of CP and control cohort.

The Student’s *t* test was used to test the difference of continuous variables.

** *p* < 0.01.

## Discussion

This is the first nationwide population-based cohort study to evaluate the association between CP exposure and pSS risk by using a matched cohort and long term follow-up period. We found a higher risk of pSS in patients with CP exposure than those who never received a diagnosis of CP. In addition, the association between CP and pSS was significant in the female group.

Recently, Lugonja et al. (2016) reported a lack of statistical significance in pSS patients with moderate/severe periodontitis. However, this study had limited sample sizes only in one hospital, making it difficult to generalize the results. Consistent with our findings, patients with pSS increased the utilization of dental services at least eight years before the definitive diagnosis with an increase in periodontal problems (Lu et al., 2014). Recently, a longitudinal prospective study demonstrated that non-surgical periodontal treatment could significantly improve salivary flow in pSS patients (Ambrósio et al., 2017). Taken together, these studies implied the positive association between periodontitis and pSS.

Our study first evaluated the risk of pSS stratified by follow-up years in multivariable Cox proportional hazard regression. The risk of pSS was significantly higher in the CP group as compared to the periodontal healthy control group. Taken together, these findings indicate that the regular oral check-up for periodontal condition may be necessary for individuals with pSS.

We hypothesized that the possible mechanism of the increased risk of CP in pSS patients may be associated with immune-inflammation and periodontal pathogens. Periodontitis is characterized by a dysregulation of the immune-inflammatory response to periodontal pathogens. Patients with pSS exhibited significantly elevated antibody levels to some predominate periodontal pathogens as compared with systemically healthy individuals (Celenligil et al., 1998) or periodontitis (Lugonja et al., 2016). It is not clear how microbiome analysis will provide meaningful data related to autoantibody levels or inflammation in general. However, overexpression of several inflammatory cytokines has been demonstrated both in pSS and CP (Barone & Colafrancesco, 2016; Darveau, 2010). Therefore, just by having CP may lead to the development of pSS without a genetic predisposition.

The strength of this study was the use of a nationwide database composed of general population in Taiwan and a matched control group that may have avoided the selection bias between CP and pSS. We adopted a frequency-matched cohort study design by using the patients with CP and adequate adjusted for sex, age, and index year. In addition, the cohort study design could confer a higher level of evidence to suggest a causal relationship rather than the case control design.

Some potential limitations should be noted before the interpretation of data. First, in Taiwan, Sjögren syndrome is recognized as a catastrophic illness covered under the NHI scheme. Patients with minor manifestations of this disease might not have applied for a catastrophic illness certificate. In this study, the ICD codes from the LHID2010 claim database might be affected by the diagnostic accuracy of this database because of the lack of data on the severity of pSS, laboratory results, and the indications for medication use. However, the regular check of the quality of claims data from all medical institutions by the NHI Administration has improved the coding accuracy and hence minimized bias due to misclassification. In addition, there are 23 medical specialties approved by government of Taiwan. Further, patients suspected of having pSS will be referred to a Rheumatology appointment for a definitive diagnosis and will receive further treatment within the NHI system.

Second, the severity of CP as a risk factor for developing pSS could not be explored. The collected data regarding the diagnosis of CP based on ICD-9 codes recorded in the NHIRD may not truly indicate the severity of periodontitis. However, to ensure the criteria of indication and quality of treatment, the NHI has established the strict guideline that only board-registered dentists can execute the dental treatment according to the strictly NHI therapeutic guidelines. Therefore, the periodontal diagnosis could reach the measurement reproducibility in Taiwan.

Third, due to this is a register-based study, the unrecognized periodontitis which did not have dental medical claims might be included in the healthy controls through the study design. By using the propensity score matching could reduce the selection bias and avoid the confounding variates.

Forth, periodontitis prevalence appears to be high even in areas with adequate access to oral health care. Therefore, perhaps misclassification bias may influence the risk estimate in this study leading to under-estimated the odds ratio of pSS for CP exposure. Our previous report has indicated that the prevalence rate of periodontal disease was estimated by lowering to 11.5% to 19.59% from NHIRD (Yu et al., 2017). Taken together, patients with CP are with increased risk for pSS in Taiwan. Finally, information on individual behaviors, such as smoking and alcohol consumption are unavailable from the database. Nevertheless, possible effects due to confounding bias from above factors might be minimized after adjusting for age, sex, income, and urbanization.

## Conclusions

The findings of this nationwide, population-based cohort study revealed a significant association between CP exposure and pSS risk. However, further prospective clinical studies on the relationship between CP and pSS are warranted.

## Supplemental Information

10.7717/peerj.5109/supp-1 Supplemental Information 1 Chronic periodontitis analysis data. The dataset was to describe the demographic data and estimate the hazard ratios of primary Sjögren syndrome.

## Additional Information and Declarations

### Data Availability

The following information was supplied regarding data availability: The data are provided in a Supplemental File.
